# A Deep Anomaly Detection System for IoT-Based Smart Buildings

**DOI:** 10.3390/s23239331

**Published:** 2023-11-22

**Authors:** Simona Cicero, Massimo Guarascio, Antonio Guerrieri, Simone Mungari

**Affiliations:** 1 Independent Researcher, 87032 Amantea, CS, Italy; simona.cicero@gmail.com; 2 ICAR-CNR, Institute for High-Performance Computing and Networking, National Research Council of Italy, Via P. Bucci 8/9C, 87036 Rende, CS, Italy; antonio.guerrieri@icar.cnr.it; 3 University of Calabria, Via P. Bucci, 87036 Rende, CS, Italy

**Keywords:** internet of things, sensor data stream, deep learning, safety, anomaly detection, industry 4.0

## Abstract

In recent years, technological advancements in sensor, communication, and data storage technologies have led to the increasingly widespread use of smart devices in different types of buildings, such as residential homes, offices, and industrial installations. The main benefit of using these devices is the possibility of enhancing different crucial aspects of life within these buildings, including energy efficiency, safety, health, and occupant comfort. In particular, the fast progress in the field of the *Internet of Things* has yielded exponential growth in the number of connected smart devices and, consequently, increased the volume of data generated and exchanged. However, traditional Cloud-Computing platforms have exhibited limitations in their capacity to handle and process the continuous data exchange, leading to the rise of new computing paradigms, such as *Edge Computing* and *Fog Computing*. In this new complex scenario, advanced *Artificial Intelligence* and *Machine Learning* can play a key role in analyzing the generated data and predicting unexpected or anomalous events, allowing for quickly setting up effective responses against these unexpected events. To the best of our knowledge, current literature lacks Deep-Learning-based approaches specifically devised for guaranteeing safety in IoT-Based Smart Buildings. For this reason, we adopt an unsupervised neural architecture for detecting anomalies, such as faults, fires, theft attempts, and more, in such contexts. In more detail, in our proposal, data from a sensor network are processed by a *Sparse U-Net* neural model. The proposed approach is lightweight, making it suitable for deployment on the edge nodes of the network, and it does not require a pre-labeled training dataset. Experimental results conducted on a real-world case study demonstrate the effectiveness of the developed solution.

## 1. Introduction

The rapid advancements in hardware, software, and communication technologies have facilitated the development of Internet-connected smart devices capable of observing and measuring data from the physical world. The term *Internet of Things* (IoT) [1] is commonly used to define a novel paradigm in which interoperable *Smart Objects* are interconnected and communicate among themselves to perform several activities (e.g., monitoring, control, optimization, and automation) [2]. Specifically, IoT technologies facilitate the creation of *Smart Buildings*. Buildings serve as fundamental yet essential components of the human living environment. The idea of Smart Buildings emerged as a result of the growing incorporation of cutting-edge IoT technology into building systems, enabling remote operation and control throughout the entire lifespan of the structures. This integration aims to enhance convenience, comfort, cost-effectiveness, and energy efficiency [3]. Today, IoT has gained extensive usage in Smart Buildings [4], representing the vanguard of architectural design: integrating sensors and actuators enables both gathering data for informed decision-making and performing intelligent actions.

Since the great diffusion of such devices, the data gathered by them has taken the form of *Big Data* [5] i.e., they are characterized by large volumes, frequency, and variety. Although the data generated by IoT networks deployed in smart buildings represent a precious source for developing intelligent tools that can enhance the perceived well-being of people while ensuring their safety, their usage entails addressing various challenges due to the nature of the data to be processed. First, the sensors employed for environment monitoring generate large data streams that need to be processed in real time to promptly detect potential unexpected behaviors or anomalies that may occur within the building, allowing for the immediate activation of appropriate countermeasures. In our reference scenario, an anomaly can result from a sensor malfunction or from an abrupt change detected in the readings of sensors within the physical environment due to an exceptional/unusual event. Examples of anomalies that can occur include fires, gas leaks, disasters, unauthorized physical access during non-working hours, and attempted theft. In this sense, detecting in time these anomalies can be crucial to take measures against possible harmful phenomena.

However, the collected data are often low-level raw data (e.g., measurements on temperature, humidity, CO${}_{2}$ levels, and air quality) that require domain expert intervention to be prepared for the analysis. Moreover, the observed behavior can naturally evolve [6]; therefore, an effective solution must be able to incrementally update itself to distinguish between true anomalies and normal changes in the monitored environment.

Given the speed and volume of data generated, recently, there has been a growing interest in developing solutions leveraging *Artificial Intelligence* (AI) and *Machine-Learning* (ML) techniques [7,8]. In particular, the approaches based on the Deep-Learning (DL) paradigm represent the state-of-the-art in several application scenarios for revealing the presence of unexpected behaviors. DL-based architectures can be used to create effective anomaly detection models by combining raw low-level data collected from sensors of various types. Deep Neural Networks (DNNs) operate hierarchically, with multiple layers of non-linear processing units organized in a hierarchical structure [9]. Each subsequent layer in the architecture produces a set of features with a higher abstraction level than the previous one. As a result, DL-based approaches are effective for deriving data abstractions and representations at various levels. They also serve as an excellent choice for analyzing raw data provided in different formats and from different sources. Finally, Deep-Learning techniques inherently leverage the availability of large volumes of data, while many traditional Machine-Learning algorithms with shallow hierarchies fail to explore and learn highly complex patterns.

However, learning accurate models requires coping with several challenging issues: *(i)* **unavailability of labeled data**, in many real-world scenarios, the training set often lacks labels and includes only normal examples for the learning phase; *(ii)* **unbalanced class distribution**, the majority of data points belong to the “normal” class, while the “anomalous” or rare class represents only a small portion of the data; and *(iii)* **zero-shot anomaly detection**, identifying anomalies that have not occurred or been observed in the past.

### 1.1. Contribution

The main objective of this work is to define a solution able to cope with all the abovementioned issues. In more detail, we are interested in learning effective and efficient detection models when labeled data are not available for the training stage. Moreover, the approach must be able to learn only from the new batches of data made available (discarding the old ones) since we want to deploy the detection models on edge devices with limited hardware resources in terms of computation and storage. To this aim, here we defined an unsupervised Deep Learning-based solution for guaranteeing safety in IoT-based Smart Buildings. The proposed model does not necessitate any prior knowledge of anomalies; moreover, our model adopts online training based on a sliding-window strategy to enhance robustness against changes in the underlying data distribution. In particular, our approach allows for detecting and reporting anomalies that can occur in a Smart Building monitored by intelligent sensors. The main contributions of this work are summarized below:

- Design and development of a neural model for identifying anomalies in large data streams from IoT sensors. Specifically, a neural architecture inspired by the Sparse U-Net (widely used in other contexts, such as cybersecurity applications [10]) has been employed. Basically, it can be figured out as an autoencoder (AE) embedding several skip connections to facilitate the network learning process and some sparse dense layers to make the AE more robust to noise. The approach is unsupervised and lightweight, making it suitable for deployment at the network’s edge.
- An extensive experimental evaluation was conducted on the test case generated using the abovementioned strategy. Numerical results demonstrate the effectiveness and efficiency of our approach.

### 1.2. Organization of the Paper

The rest of the paper is organized as follows: Section 2 reviews the state-of-the-art methods for detecting anomalies in Smart Building; Section 3 illustrates the unsupervised neural architecture adopted in this work and showcases the detection approach used to reveal the presence of unexpected behaviors; Section 4 describes the considered case study, while Section 5 details the experimental evaluation conducted on the introduced case. Finally, Section 6 concludes the paper and outlines future research directions.

## 2. Related Works

IoT systems produce massive quantities of data. Thus, identifying abnormal observations in data streams is not trivial. Many models have been proposed over the years exploiting different frameworks. In Shahraki et al. [11], the authors proposed TONTA: an online model able to identify abrupt changes. For this purpose, the proposed model aims to compare different network traffic trends by exploiting time-series data gathered from the IoT architecture. In particular, the distance between two sequences is computed, and if the result is above a specific threshold, a change point is detected and notified. This approach can be easily adopted in scenarios with limited computational and storage resources.

Some other different approaches in the literature are based on the use of Machine/Deep-Learning techniques since they are successfully adopted in several scenarios due to their capabilities of capturing patterns by analyzing historical data. For example, within the context of *Internet of Vehicles* (IoV), in [12], the authors exploit a single LSTM by combining temporal and data dimensions in the input. The model’s output is then compared to the actual data to identify possible anomalies. A different solution exploiting DL approaches is proposed by Gao et al. [13], which is used for identifying anomalies in IoT time-series data. The proposed model, TSMAE, uses a sequence-to-sequence architecture based on LSTMs for reproducing the input to the output. Moreover, it is empowered with a memory module [14] to enhance the computation by combining information extracted from old data with current observations.

Recently, distributed frameworks, such as federated machine/deep-learning models [15,16,17] and *Edge Computing* [18], have been investigated for developing efficient solutions for revealing anomalies in different applications. In this setting, each network node can process its local data without the need to send data to an external processing unit (e.g., the Cloud). An example is READ-IoT [19], which adopts a bifold architecture: the first module, based on logic rules, detects whether there is an anomaly following some predefined thresholds (such as temperature, humidity and so on); while the second module, based on ML models, is used to *double-check* the previous output (*refinement*). It is worth noting that the main limitation of this approach is that it is wired to specific scenarios, and it is not a general-purpose model. In [20], the authors devised a model for anomaly detection in edge computing, which introduces a novel framework encompassing data compression and a moth flame swarm optimization algorithm to route the data among the network efficiently. Finally, an LSTM is used in a supervised framework to predict whether the observation is anomalous. Alas, in real-world scenarios, it is unlikely to have labeled anomalies. For this reason, unsupervised approaches are frequently preferred to supervised ones. To overcome this limitation, our proposed model detects anomalies in an unsupervised fashion. However, to the best of our knowledge, our approach is the first tentative to combine unsupervised deep neural models with a sliding-window strategy to reveal the presence of unexpected behaviors to guarantee safety in IoT-Based Smart Buildings.

## 3. Proposed Approach

This section illustrates the Deep Learning-based solution designed to identify anomalous behaviors within Smart Buildings. Our detection model adopts an unsupervised approach and incorporates a preprocessing procedure to enhance its efficacy.

The main benefit of employing an unsupervised learning scheme relies on its capacity to trigger alerts for previously unseen anomalies. This is a common scenario in sensor-monitored environments, as incidents and safety issues are frequently undocumented and unknown beforehand.

### 3.1. Neural Detector Architecture

The underlying idea of the proposed solution is to employ an *encoder-decoder* architecture trained on data collected from a sensor network deployed in a monitored environment. Essentially, an *autoencoder (AE)* is an unsupervised neural network model (i.e., trained without any prior knowledge about the nature of normal/anomalous behavior) that performs two main operations: first, it compresses the input data (i.e., a set of statistics computed from sensor measurements at specific time intervals) into a *latent space*, and subsequently, it attempts to reconstruct the original information provided as input to the model.

In the considered application scenario, the model is trained solely on normal data, i.e., data in which anomalies are not present. The fundamental insight is that input data describing normal behaviors should be mostly reconstructed accurately by the autoencoder. In other words, the encoding/decoding phases should not introduce significant distortion in the output. Conversely, outliers and anomalies in the input should lead the AE to generate a “*deviant*” output. Basically, the approach is unsupervised because the neural network learns the identity function between input and output (without considering the anomaly label). In an operational scenario, the model is trained on a limited number of normal examples selected by the expert, and then, as soon as new tuples for the learning stage are made available, the ones more likely “normal” are used to update the model. The detection of the anomalies is performed based on the divergence between the input and the model output.

The use of reconstruction error as a measure of outliers to discover anomalous behaviors has already been proposed in the literature. However, the application of unsupervised techniques (particularly encoder-decoder architectures) to detect critical situations in industrial and smart building environments is currently the subject of study. As discussed in [21,22], autoencoders are regarded as a valid solution to effectively summarize the main information of a given input into a low-dimensional representation. In essence, these neural network models aim to produce an output that closely duplicates the provided input.

**Figure 1:**
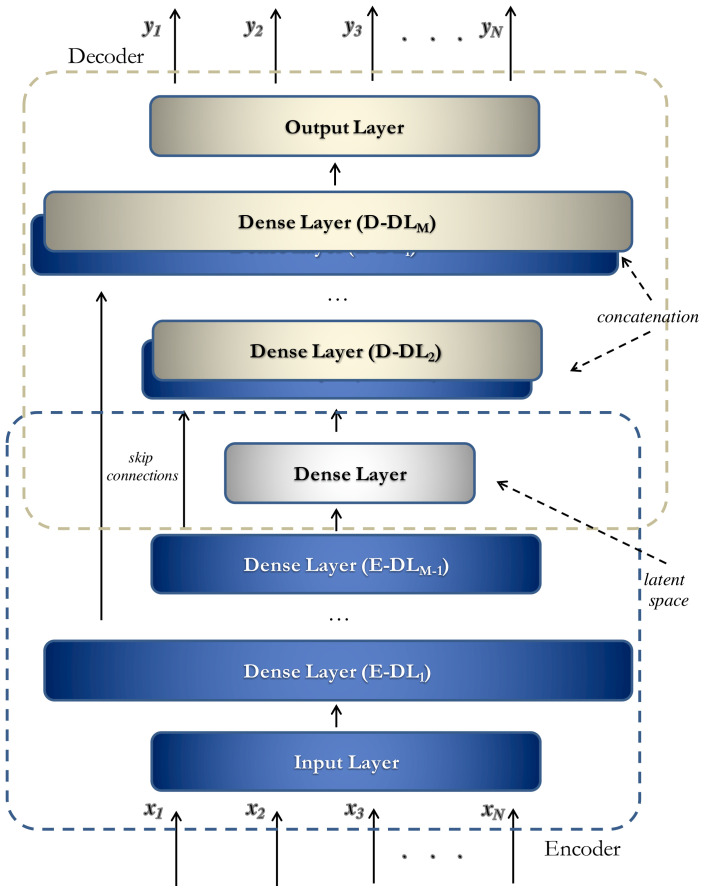
Neural architecture (Sparse U-Net) used to detect anomalous behaviors in our approach.

In this work, we adopted the architecture depicted in Figure 1 [10]. Basically, it consists of two main components referred to as the *Encoder* and the *Decoder*.

Let $x=\{x_{1},\cdots ,x_{N}\}$ be a set of numerical features (in our scenario, a set of statistics computed from sensor data within a specific time interval). The first subnetwork’s goal is to map the input data into a latent space (*encoding*), i.e., to learn a function z=enc(x). The second subnetwork provides the overall network output y=dec(z) by reconstructing the input from the features extracted by the encoder (*decoding*). Gradient descent is employed to learn the model weights by minimizing an appropriate loss function. The Mean Squared Error (MSE) [23], defined as $MSE=\frac{1}{N}\sum_{i=1}^{N}{\left(x_{i}-y_{i}\right)}^{2}$, is used for the learning phase.

Please note that the architecture in Figure 1 exhibits two main differences compared to a standard encoder-decoder model:

- *Skip Connections*: These connections allow the layers of a neural network to be connected in a way that enables a direct flow of information from one layer to another, bypassing one or more intermediate layers and, in this way, preserving information and gradients [24]. Skip connections allow the construction of much deeper neural networks without suffering from performance or training issues. This is particularly useful because deeper neural networks can capture more complex data representations. Moreover, they enable neural networks to learn residual differences between input data and the predicted data. Hence, skip connections enhance the model’s predictive performance and reduce the number of iterations required for the convergence of the learning algorithm.
- *Hybrid Approach*: The architecture incorporates the use of “*Sparse Dense Layers*” to make the autoencoder more robust to noise, particularly because the anomalies to be identified often exhibit slight differences from normal behaviors. Sparse Dense Layers used in our solutions fall within the Sparse-AE framework. In this scenario, a Sparse Dense Layer is essentially a dense layer with a significantly larger number of neurons compared to the size of its input. However, what makes it “sparse” is that the learning process actively encourages sparsity in the activations within this layer. This means that only a subset of neurons is encouraged to be active, with non-zero activations for a given input. The primary purpose of this design is to reduce the complexity of the representations learned by the network. By promoting sparsity in the activations, the Sparse Dense Layer effectively learns a more concise and efficient representation of the input data. This can be particularly advantageous in scenarios where data dimensionality reduction or feature selection is desired. The architecture’s Sparse Dense Layers are placed in the first layer of the encoder and the last layer of the decoder. Both the encoder and the decoder consist of *M* hidden layers, resulting in a symmetrical architecture.

In more detail, the use of skip connections simplifies the learning process by providing, as input to each layer of the decoder (*D*-$DL_{i}$), except for the shared latent space, both the previous layer (*D*-$DL_{i-1}$) and the corresponding layer of the encoder (*E*-$DL_{M-i+1}$). As for the Sparse Layers, they are employed to generate a greater number of discriminative features, allowing for the extraction of a more representative latent space.

### 3.2. Detection Protocol

**Figure 2:**
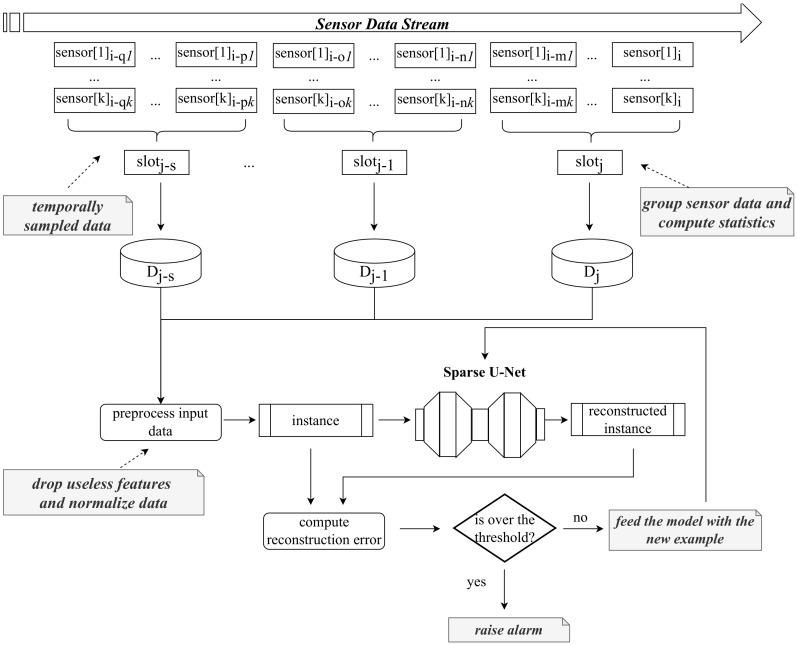
Detection mechanism used to reveal the presence of anomalies.

Figure 2 illustrates the anomaly detection process. Without loss of generality, we assume to monitor an “*infinite data stream*”, i.e., data produced by *k* IoT devices continuously feeding the detection mechanism.

At each predefined time interval (corresponding to a “*time slot*” in the figure), a set of descriptive statistics is computed to represent the state of the environment being monitored during that specific time window. This approach offers several advantages, most notably the ability to carry out this process without overloading network traffic and using limited computational resources. This efficiency is achieved because, for both the learning and inference phases, it is only necessary to store aggregated statistics. These statistics are generated based on the measurements provided by the sensors and include metrics such as the *minimum*, *average*, *maximum*, different percentile values, and more.

The data to be processed by the Sparse-AE model are preprocessed to improve the effectiveness of the approach. This preprocessing phase includes normalizing the data within the range of [−1,1], achieved through a MinMax strategy. Additionally, any irrelevant features, such as those exhibiting minimal variability, are dropped from the dataset. This preprocessing is a crucial step performed consistently in both the learning phase and the deployment stage.

The heart of the process lies in utilizing an autoencoder that has been pre-trained on normal data verified by an expert. This autoencoder is used to reproduce the computed statistics, and subsequently, the reconstruction error for the current example is calculated as the Mean Squared Error between the original input data, denoted as *x*, and the autoencoder’s output denoted as *y*. The core of the detection system is included in this phase: if the reconstruction error falls below a predefined “*outlierness threshold*”, the current data are labeled as “normal”. This labeled data then contributes to updating the detection model, ensuring that it remains current and capable of accurately capturing the evolving state of the monitored environment. On the other hand, if the reconstruction error exceeds the established threshold, the system triggers an alert, signaling a potential anomaly or deviance in the environment. This approach not only allows for efficient real-time monitoring but also provides the flexibility to adapt to changing conditions.

## 4. Case Study

The case study for testing the proposed approach has been obtained by altering and injecting anomalies in the dataset described in [25], and that can be found on GitHub (Occupancy-detection-data—https://github.com/LuisM78/Occupancy-detection-data Last seen on 23 October 2023). The dataset has been obtained by gathering experimental measurements from different types of sensors necessary for occupancy detection. Specifically, the data were obtained by monitoring an office room with approximate dimensions of 5.85 m × 3.50 m × 3.53 m (L × W × H) using specific sensors to real-time monitor (every minute) the following parameters: temperature, humidity, light, and CO${}_{2}$ levels. The devices used for data collection are listed below:

- A microcontroller for preprocessing the data.
- A ZigBee radio is connected to the microcontroller for collecting data from the sensors and transmitting the information to a recording station.
- A digital camera to determine room occupancy.

Collected data were made available in the form of three datasets whose main characteristics are listed in Table 1. In particular, each dataset consists of 7 fields, also called features, which are all numeric except the last one, which is binary: *Date*, *Temperature*, *Humidity*, *Light*, *CO_2_*, *HumidityRatio*, and *Occupancy*.

However, since the training dataset consists mainly of measurements recorded with the door closed during the occupied state, we extract a sample from Testing_2 equal to the first 50% of the data (i.e., 4876 tuples) and add it to the original training set of 8143 tuples. In this way, we aim to feed the neural network with a wider variety of possible normal cases during the learning phase. The new training set will thus contain 13,019 tuples, while Testing_2 will only contain the remaining 4876 tuples. The new datasets are named, respectively, Training_1_plus, Testing_1, and Testing_2_sampled and are highlighted in Table 2.

### Injecting Synthetic Anomalies

Here, we describe the protocol adopted to insert unexpected behaviors in both the test cases introduced above, namely Testing_1 and Testing_2_sampled. Let *i* be the i-th feature randomly selected from the list of features to be considered within a generic test set. Three types of anomalies have been generated:

- *Peak Anomalies.* In this case, we replace the actual value $x_{i}$ with $anomaly(x_{i})$. The anomaly is computed using the following formula:
  $$anomaly\left(x_{i}\right)=\mu_{i}\pm \alpha \cdot \sigma_{i}$$
  where α is a real number sampled from the interval [2,4], $\mu_{i}$ is the mean value of the feature *i*, and $\sigma_{i}$ represents its variance.
- *Sensor Fault Anomalies*. The i−th feature of $x_{i}$ is set to zero to simulate the breakdown of the corresponding sensor. It is assumed that each fault generates a 15-min window in which the sensor does not detect any measurements, meaning it consistently records a null value.
- *Expert-Induced Anomalies*. These are anomalies conveniently added by a domain expert that simulate three different scenarios: *(i)* a fire, *(ii)* a window left open in the room, and *(iii)* people staying in the room at night. These kinds of anomalies involve changes in different features together since a real event in the environment is simulated (e.g., in the case of fire, the CO${}_{2}$ dramatically increases together with the temperature, while the humidity decreases; in the case of a window opened, the CO${}_{2}$ slowly decreases together with the temperature, while the humidity increases).

The number of anomalies injected depends on the type and, specifically, the following were injected:

- 100 peak anomalies (corresponding to 100 modified tuples);
- 25 sensor fault anomalies (i.e., 25·15=375 modified tuples);
- 10 expert-induced anomalies (equal to 160 modified tuples).

After inserting the anomalies, the data in each test set was grouped into time slots following the approach described in Section 3.2 in order to calculate the descriptive statistics to be provided as input to the neural model. In our experimentation, the statistics were computed by considering a time window with a duration of 5 min.

## 5. Experimental Section

In this section, we describe a set of experiments aimed at evaluating the detection capabilities of our approach against the case study described above. The experiments were conducted to: (*i*) compare the accuracy of our neural model with respect to a baseline model, (*ii*) analyze the sensitiveness of the approach in terms of detection capabilities with respect to the threshold parameter, and finally (*iii*) evaluate the convergence rate of the proposed model.

### 5.1. Parameter Settings and Evaluation Metrics

As discussed in Section 3, the neural architecture adopted in our solution approach includes different layers: the sparse layer of the encoder is composed of 256 neurons, while the other ones are 24 and 16, respectively. The latent space includes 8 neurons. The decoder layer is built symmetrically. Each layer is equipped with a *ReLU* activation function [26] except for the output layer, which was equipped with a *linear* activation while Adam is used as optimizer [27]. The parameters used for the training phase are summarized below in Table 3.

To fully evaluate our model, we compared the accuracy performances with a Deep Autoencoder (DAE) model. The main differences between the two architectures are: (*i*) DAE does not include skip connections, and (*ii*) the sparse layer has been replaced with a linear layer with 28 neurons.

A key parameter of the proposed approach is the reconstruction error threshold, which allows for discriminating between normal behaviors and anomalies. In our evaluation, we considered three different threshold values estimated by computing the reconstruction error against the training set and sorting the obtained values:

- *98th percentile*. The threshold is the 98th percentile of the training reconstruction errors;
- *max_value*. The threshold is the maximum value among the reconstruction errors of the training set data;
- *max + tolerance*. It is computed according to the following formula: max+tolerance=max_value+(max_value−98thpercentile)

The performances of our approach have been assessed using well-known quality metrics commonly used in unbalanced scenarios. For this evaluation, we define TP as the count of correctly classified positive cases, FP as the count of negative cases incorrectly classified as positive, FN as the count of positive cases incorrectly classified as negative, and TN as the count of correctly classified negative cases.

Leveraging these values, we can calculate the following metrics [28]:

- *Accuracy*: defined as the fraction of cases correctly classified, i.e., $\frac{TP+TN}{TP+FP+FN+TN}$;
- *Precision* and *Recall*: metrics employed for assessing a system’s ability to detect anomalies, as they offer a measure of accuracy in identifying anomalies while minimizing false alarms. Specifically, Precision is defined as $\frac{TP}{TP+FP}$, while Recall as $\frac{TP}{TP+FN}$;
- *F-Measure*: summarizes the model performance and computed as the harmonic mean of Precision and Recall.

Lastly, to perform experiments, we used a machine with 16 GB RAM, an AMD Ryzen 7 5700U CPU @4.30GHz, and a 1TB SSD drive.

### 5.2. Quantitative Evaluation: Comparison with the Baseline and Sensitivity Analysis

Although all the measures are reported in the following tables, we focused the analysis on the F−Measure as it effectively summarizes the overall performances of the model with a single value and Accuracy, which provides a helicopter view of the performances. Moreover, the performance values obtained by ranging different anomaly thresholds are shown.

We can observe that the proposed model outperforms the baseline on both the test cases in terms of F−Measure and Accuracy (results in Table 4, Table 5, Table 6, Table 7, Table 8 and Table 9), except for the *sensor fault anomalies* in Testing_1.

Regarding the sensitivity analysis, we consider mainly the F−Measure.

Examining all the tables, we can note that for both neural architectures, as the threshold increases, the Precision also increases, while Recall decreases, and vice versa. This allows us to state that by adjusting the threshold value, it is possible to obtain a more precise system or one with higher Recall, depending on the application context: if the goal is to have a more accurate system, a higher threshold is required, while if the system still needs to detect as many anomalies as possible, even at the cost of raising more false alarms, then the threshold value should be lowered. This analysis aimed to determine which threshold allows for capturing the highest number of anomalies while simultaneously limiting the number of false alarms. We can observe that for Testing_1, the best threshold is the 98th percentile while for Testing_2_sampled is the max_value. This is mainly due to how the Training_1_plus is composed. Indeed, it contains more tuples with the door closed (as in the Testing_1 dataset) than with the door opened (as in the Testing_2_sampled dataset).

**Figure 3:**
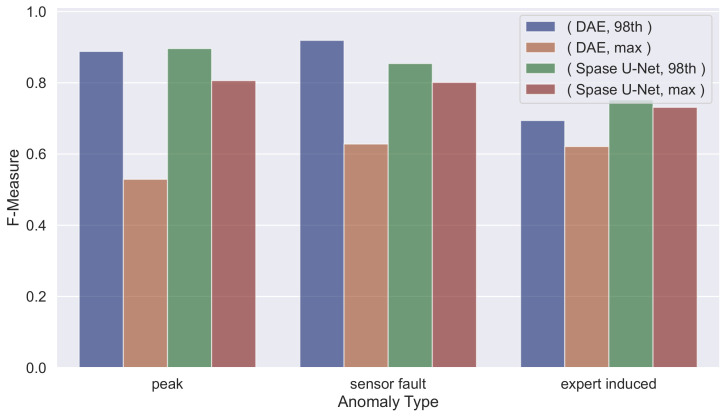
F-Measure comparison varying different settings per anomaly type on *Testing_1*.

**Figure 4:**
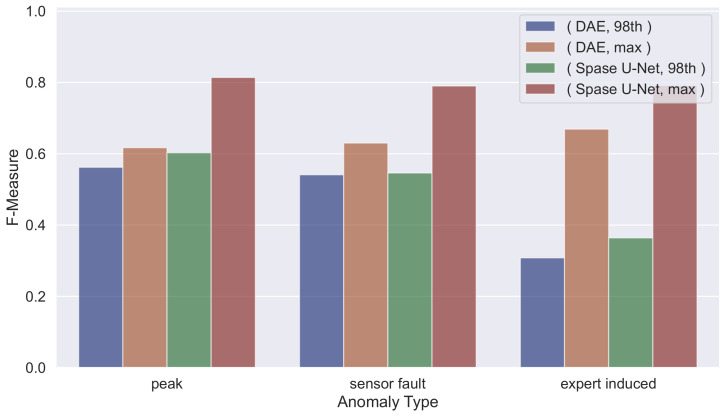
F-Measure comparison varying different settings per anomaly type on *Testing_2_sampled*.

The obtained results are better compared in Figure 3 and Figure 4, in which we highlight, for *Testing_1* and *Testing_2_sampled*, a comparison on the F-Measure using the best-performing thresholds (i.e., *98th percentile* and *max value*). In particular, in such figures, we show the different F-Measure values given by the used *Deep Autoencoder* and *Sparse U-Net* models by varying the anomaly type. These charts provide a helicopter view of the overall performances achieved in different settings and could allow any expert to choose the best-needed threshold according to the specific scenario.

### 5.3. Convergence

The objective of the following analysis is to verify whether the Sparse U-Net architecture allows for faster convergence of the learning algorithm. In detail, the behavior of the loss function on the training set for the two architectures was compared regarding the number of epochs.

**Figure 5:**
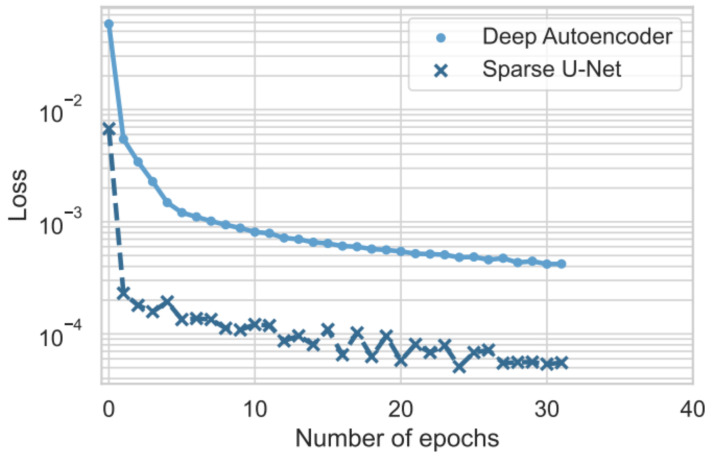
Deep Autoencoder and Sparse U-Net loss functions trend in logarithmic scale.

It is worth noting that, at the first iteration, the loss value of the Sparse U-Net is lower by 0.05 compared to the loss value of the DAE. This demonstrates the benefit of adopting skip connections and sparse layers in the early stages of training. This behavior is even more evident in Figure 5, which presents the comparison of the loss functions on a logarithmic scale.

Indeed, we can note that the proposed model converges much faster than the baseline model. However, the rapid convergence of the loss does not lead to overfitting phenomena. As shown earlier, the Sparse U-Net exhibits superior predictive performance compared to the baseline in all the evaluated test scenarios except for one.

Finally, we measured the time required for both the training and inference phases. Basically, in the learning stage, the model is able to process the entire dataset in 3 s. (*Time per epoch*) and a single data batch in 3 ms, whereas as regards the inference time (*per tuple*), the model requires less than 1 ms to yield the output for a single input. This demonstrates the efficiency of our solution and the possibility of deploying it on the edge of the network (lightweight).

## 6. Conclusions

IoT technology is becoming increasingly pervasive in people’s lives. Its ability to enable the realization of Smart Buildings is increasing the willingness of the buildings’ inhabitants to have IoT devices spread in their homes since they can enhance convenience, comfort, cost-effectiveness, and energy efficiency in building environments. Based on the January 2023 update from the Global IoT Enterprise Spending Dashboard by IoT Analytics, the overall cost of enterprise IoT in 2022 saw a significant growth of 21.5% (IoT Analytics—https://iot-analytics.com/iot-market-size/ Last seen on 23 October 2023), highlighting the increasing interest in these technologies. IoT sensors deployed in Smart Buildings generate large amounts of data that no longer require physical transportation for processing and analysis. Meanwhile, new technologies based on Artificial Intelligence enable the detection of anomalies almost in real time, allowing for prompt responses to them.

In this work, a Machine-Learning-based approach has been defined for the analysis of data from a sensor network to detect anomalies related to sensor malfunctions or exceptional events such as *fires*, *gas leaks*, and *intrusion attempts*.

In more detail, a specific Deep-Learning architecture has been developed for identifying unexpected behaviors, which is an Autoencoder that integrates skip connections to facilitate the network’s learning process and sparse dense layers to make the AE more robust to noise. This model can be trained without the need for previously labeled data. Therefore, the approach is unsupervised and lightweight, making it suitable for use directly on the network’s edge nodes. The experimentation conducted on a real case study demonstrates the quality of the proposed approach, which achieves an F-Measure improvement until the 20% compared to the baseline model, also lowering the convergence time.

### Challenges and Opportunities

Although the results are encouraging and significant, some challenges remain open. First, the presence of changes in data distributions due to normal situations (e.g., the increase in temperature during the transition from winter to spring) could affect the predictive capacity of the model (*Concept Drift*). Basically, the prompt detection of these changes can allow for updating the model to reduce the risk of raising false alarms. Different approaches have been proposed to tackle this issue. One common approach is to implement real-time monitoring systems that continuously collect and analyze data to identify *abrupt* or *gradual* deviations from the expected patterns. Another strategy is to employ anomaly detection methods that highlight unusual patterns or outliers in the data. These anomalies may signify changes in the underlying processes, warranting further investigation. Machine-learning models, such as neural networks and decision trees, can be trained to identify these changes. When the concept drift detection methods fail, the use of *Ensemble Learning* approaches can represent an effective tool to mitigate this problem, as different models learned in different time intervals could be combined to gradually adapt to the new state while maintaining the memory of the previous state. Hence, the learned models can be combined using trainable or not-trainable functions to weigh the importance of each model according to different criteria (e.g., more recent models could influence the response of the ensemble more than the old ones). Additionally, the possibility of combining information from multiple edge nodes using Federated-Learning (FL) tools [29,30] (via the exchange of DL models only) can be harnessed to leverage the collaborative capacity of these new distributed systems, making them even more effective [31]. Moreover, these methods play a key role in all those scenarios where data cannot be moved in a single place (e.g., due to privacy issues) to perform traditional centralized learning. Essentially, FL comprises a range of methods and techniques designed to facilitate the training of machine-learning models in a decentralized manner. Typically, this decentralized learning takes place on edge devices or servers that store data. By promoting collaboration among various stakeholders, FL enables the development of robust and efficient predictive models without the necessity of centralized data sharing. However, it is worth noting that several FL frameworks involve multiple rounds of communication between devices and the central server, resulting in heightened communication overheads. This represents a main limitation; therefore, we are interested in investigating current and new protocols and strategies for efficient learning federated models in our scenario. As future works, we want to also extend the experimentation by considering other approaches leveraged in different IoT-based scenarios to highlight the quality of our technique. Finally, another relevant aspect concerns the defense of these models, whose shared in a distributed setting could be subject to different forms of cyberattacks [32,33]. For instance, they can be targeted through techniques known as data poisoning. In this scenario, an adversary intentionally injects malicious data into the training dataset used to train a DL model. This poisoned data can skew the model’s understanding of normal behavior, making it less effective at identifying anomalies. Moreover, attackers can tamper with the weights and parameters of a neural network to achieve specific outcomes for particular inputs. By manipulating these weights, they can make the network produce desired outputs, allowing for the exfiltration of sensitive information. This phenomenon is often referred to as *data leakage*. In essence, these attack methods can render deep-learning models vulnerable to adversaries seeking to evade detection and compromise security systems.

## Figures and Tables

**Table 1:** Initial datasets.

Dataset	Features	Number of Tuples	Further information
Training	7	8143	Measurements mainly obtained with the closed door while the room is occupied
Testing_1	7	2665	Measurements mainly obtained with the closed door while the room is occupied
Testing_2	7	9752	Measurements mainly obtained with the opened door while the room is occupied

**Table 2:** Considered datasets for experimental evaluation.

Dataset	Features	Number of Tuples	Further information
Training_1_plus	7	13,019	Measurements obtained both with the closed and the opened door while the room is occupied
Testing_1	7	2665	Measurements mainly obtained with the closed door while the room is occupied
Testing_2_sampled	7	4876	Measurements mainly obtained with the opened door while the room is occupied

**Table 3:** Training parameters of the Sparse U-Net.

Parameters	Values
batch_size	16
num_epoch	32
optimizer	adam
loss	mse

**Table 4:** Experimental results for Testing_1 dataset altered with *peak anomalies*. In bold and italics are reported the best results for the Sparse U-Net and the baseline, respectively.

Neural Model	Threshold	Accuracy	Precision	Recall	F-Measure
Deep Autoencoder ( *baseline* )	*98th percentile*	*0.956*	0.801	*0.996*	*0.888*
	*max value*	0.889	*1.000*	0.359	0.529
	*max + tolerance*	0.868	*1.000*	0.242	0.390
Sparse U-Net ( *Proposed Model* )	*98th percentile*	**0.960**	0.814	**0.996**	**0.896**
	*max value*	0.943	0.984	0.682	0.806
	*max + tolerance*	0.926	**1.000**	0.571	0.727

**Table 5:** Experimental results for Testing_1 dataset altered with *sensor fault anomalies*. In bold and italics are reported the best results for the Sparse U-Net and the baseline, respectively.

Neural Model	Threshold	Accuracy	Precision	Recall	F-Measure
Deep Autoencoder ( *baseline* )	*98th percentile*	*0.969*	0.851	*1.000*	*0.919*
	*max value*	0.903	*1.000*	0.458	0.628
	*max + tolerance*	0.885	*1.000*	0.352	0.521
Sparse U-Net ( *Proposed Model* )	*98th percentile*	**0.948**	0.850	**0.859**	**0.854**
	*max value*	0.940	0.985	0.675	0.801
	*max + tolerance*	0.893	**1.000**	0.399	0.570

**Table 6:** Experimental results for Testing_1 dataset altered with *expert-induced anomalies*. In bold and italics are reported the best results for the Sparse U-Net and the baseline, respectively.

Neural Model	Threshold	Accuracy	Precision	Recall	F-Measure
Deep Autoencoder ( *baseline* )	*98th percentile*	0.944	0.586	*0.850*	*0.694*
	*max value*	*0.959*	*1.000*	0.450	0.621
	*max + tolerance*	0.948	*1.000*	0.305	0.467
Sparse U-Net ( *Proposed Model* )	*98th percentile*	0.955	0.645	**0.900**	**0.752**
	*max value*	**0.967**	0.959	0.590	0.731
	*max + tolerance*	0.959	**1.000**	0.460	0.630

**Table 7:** Experimental results for Testing_2_sampled dataset altered with *peak anomalies*. In bold and italics are reported the best results for the Sparse U-Net and the baseline, respectively.

Neural Model	Threshold	Accuracy	Precision	Recall	F-Measure
Deep Autoencoder ( *baseline* )	*98th percentile*	0.852	0.395	*0.973*	0.562
	*max value*	*0.945*	0.986	0.449	*0.617*
	*max + tolerance*	0.927	*1.000*	0.249	0.399
Sparse U-Net ( *Proposed Model* )	*98th percentile*	0.875	0.438	**0.966**	0.603
	*max value*	**0.968**	0.952	0.711	**0.814**
	*max + tolerance*	0.961	**1.000**	0.604	0.753

**Table 8:** Experimental results for Testing_2_sampled dataset altered with *sensor fault anomalies*. In bold and italics are reported the best results for the Sparse U-Net and the baseline, respectively.

Neural Model	Threshold	Accuracy	Precision	Recall	F-Measure
Deep Autoencoder ( *baseline* )	*98th percentile*	0.846	0.381	*0.928*	0.541
	*max value*	*0.947*	0.987	0.463	*0.630*
	*max + tolerance*	0.939	*1.000*	0.371	0.541
Sparse U-Net ( *Proposed Model* )	*98th percentile*	0.864	0.405	**0.838**	0.546
	*max value*	**0.965**	0.966	0.667	**0.790**
	*max + tolerance*	0.943	**1.000**	0.415	0.586

**Table 9:** Experimental results for Testing_2_sampled dataset altered with *expert-induced anomalies*. In bold and italics are reported the best results for the Sparse U-Net and the baseline, respectively.

Neural Model	Threshold	Accuracy	Precision	Recall	F-Measure
Deep Autoencoder ( *baseline* )	*98th percentile*	0.831	0.185	*0.915*	0.308
	*max value*	*0.979*	0.971	0.510	*0.669*
	*max + tolerance*	0.974	*1.000*	0.370	0.540
Sparse U-Net ( *Proposed Model* )	*98th percentile*	0.862	0.225	**0.960**	0.364
	*max value*	**0.985**	0.893	0.710	**0.791**
	*max + tolerance*	0.980	**1.000**	0.510	0.675

## Data Availability

The original data used in this work are available at https://github.com/LuisM78/Occupancy-detection-data (accessed on 23 October 2023).
